# Detection and multilocus genotyping of *Giardia duodenalis* in dogs in Sichuan province, China

**DOI:** 10.1051/parasite/2017032

**Published:** 2017-08-03

**Authors:** Yue Zhang, Zhijun Zhong, Lei Deng, Maoqing Wang, Wei Li, Chao Gong, Hualin Fu, Suizhong Cao, Xianpeng Shi, Kongju Wu, Guangneng Peng

**Affiliations:** 1 Key Laboratory of Animal Disease and Human Health of Sichuan Province, College of Veterinary Medicine, Sichuan Agricultural University Sichuan Province 611130 P.R. China; 2 Sichuan Agricultural University, Teaching Animal Hospital, Yaan Sichuan Province 625000 P.R. China; 3 Chengdu Research Base of Giant Panda Breeding Chengdu Sichuan Province 611130 P.R. China

**Keywords:** *Giardia*, Protozoan, Dog, China, PCR

## Abstract

*Giardia duodenalis* (also known as *G. intestinalis*) is a flagellated protozoan that parasitizes the small intestine and is a common causal agent of zoonotic infections in humans and animals. To assess the genetic diversity and zoonotic transmission potential of *G. duodenalis* in stray dogs, 159 fecal specimens were collected from dogs in Chengdu, Yaan, and Leshan in Sichuan province, China. Of the 159 fecal samples from stray dogs, 18 (11.3%) were *G. duodenalis*-positive based on nested PCR amplification of the beta giardin (*bg*) gene, and the occurrence varied from 1.8% to 35% in different cities. Dog-specific assemblages C (*n* = 9) and D (*n* = 9) were identified. The glutamate dehydrogenase (*gdh*) and triosephosphate isomerase (*tpi*) genes of all *bg*-positive isolates were characterized. A total of 16 and 8 isolates were positive for the *gdh* and *tpi* genes, respectively. Two novel sequences of the *bg* locus were detected among genetic assemblage D isolates, and one novel *gdh* sequence and four novel *tpi* sequences were identified among genetic assemblage C isolates. Mixed infections of assemblages C and D were also detected. Assemblages A and B, which have high zoonotic potential, were not detected. Our results show that *G. duodenalis* is prevalent and a cause of diarrhea in dogs in Sichuan province, China.

## Abstract (fr)

*Giardia duodenalis* (également connu sous le nom de *G. intestinalis*), est un protozoaire flagellé qui parasite l’intestin grêle et un agent causal commun d’infections zoonotiques chez les humains et les animaux. Pour évaluer la diversité génétique et le potentiel de transmission zoonotique de *G. duodenalis* chez les chiens errants, 159 échantillons de matières fécales ont été prélevés chez des chiens à Chengdu, Yaan et Leshan, dans la province du Sichuan, en Chine. Sur 159 échantillons de matières fécales provenant de chiens errants, 18 (11.3 %) étaient positifs pour *G. duodenalis* sur la base de l’amplification par PCR imbriquée du gène de la bêta giardine (*bg*), et l’occurrence variait de 1.8 % à 35 % dans différentes villes. Les assemblages spécifiques au chien C (*n* = 9) et D (*n* = 9) ont été identifiés. Le gène de la glutamate déshydrogénase (*gdh*) et le triosephosphate isomérase (*tpi*) de tous les isolats *bg*-positifs ont été caractérisés. Au total, 16 et 8 isolats étaient positifs pour les gènes *gdh* et *tpi*, respectivement. Deux nouvelles séquences du locus *bg* ont été détectées parmi les isolats d’assemblage génétique D, et une nouvelle séquence *gdh* et quatre nouvelles séquences *tpi* ont été identifiées parmi les isolats d’assemblage génétique C. Des infections mixtes des assemblages C et D ont également été détectées. Les assemblages A et B, qui ont un potentiel zoonotique élevé, n’ont pas été détectés. Nos résultats montrent que *G. duodenalis* est répandu et une cause de diarrhées chez les chiens dans la province du Sichuan, en Chine.

## Introduction

*Giardia duodenalis*, also known as *G. intestinalis* or *G. lamblia*, is an important zoonotic intestinal parasite that infects humans and a variety of domestic and wild animals [18]. *G. duodenalis* has high potential for zoonotic transmission via water or feces; diarrhea is a major clinical sign of infection. In humans, *G. duodenalis* can infect immunocompromised hosts [21], such as AIDS patients, and can be life-threatening. *G. duodenalis* has been reported in livestock [12, 16, 26], wildlife [13, 20], and companion animals [10, 11, 15, 27]; it infects numerous mammalian species, including humans and species that are closely connected with humans. It is distributed worldwide and poses a threat to public health.

There are eight known genotypes (A–H) of *G. duodenalis* [7]. Assemblages A and B have zoonotic potential and can infect humans [5], cattle [28], sheep [26], dogs [19], and cats [2]. Specifically, subgenotypes of assemblages A (subtypes A1, A2, A3, and A4) and B (subtypes B1 and B4) are associated with human infections [5]. Hence, assemblages A and B are considered zoonotic genotypes. Genetic assemblages C–H have been reported in specific hosts. Assemblages C and D are observed in canines [10, 11, 27]. Assemblage E has been reported in cattle [12]. Assemblage F is specific to cats, pigs, and cetaceans [1, 15, 20], and assemblage G is specific to mice and rats [30]. Assemblage H was reported in the grey seal and gulls [9].

Many recent epidemiological studies have reported *G. duodenalis* infections in dogs in China [10, 11, 19, 27], Japan [8], and Brazil [4]. In Heilongjiang and Shanghai, China [11, 27], zoonotic genetic assemblages A and B have been observed in pet dogs. Stray dogs, considered important vehicles, have played a significant role in transmission to humans in developing countries. However, little is known about the prevalence of *G. duodenalis* in stray dogs in China. In developing countries, stray dogs are common and have a wide distribution. In this study, nested PCR was used to amplify the beta giardin (*bg*), glutamate dehydrogenase (*gdh*), and triosephosphate isomerase (*tpi*) loci to investigate the prevalence and genotypes of *G. duodenalis* in stray dogs in Sichuan province, China.

## Materials and methods

### Fecal specimen collection

From November 2016 to January 2017, 159 fecal specimens were collected from stray dogs in shelters in Chengdu, Yaan, and Leshan cities in Sichuan province, China. Forty specimens whose hosts had diarrhea were obtained from Chengdu. Sixty-three and 56 specimens whose hosts did not exhibit diarrhea were obtained from Yaan and Leshan, respectively. All dogs were stray and abandoned and were housed in shelters for at least 2 months. Fecal samples were collected, numbered in plastic containers, and transported to our laboratory in ice packs on the day of collection. These fecal specimens were stored in a 4 °C refrigerator.

### DNA extraction

Fecal specimens (50–100 mg) were removed from each plastic container. DNA was extracted directly from fecal samples using an E.Z.N.A. Stool DNA Kit (Omega Biotek, Norcross, GA, USA), according to the protocol recommended by the manufacturer. DNA samples were stored at −20 °C until use for PCR.

### PCR amplification

*G. duodenalis* was detected by nested PCR amplification of an approximately 530-bp fragment of the *bg* locus. The *bg*-positive products were further characterized by amplification of *gdh* and *tpi*. The primers for PCR amplification and annealing temperatures for the three genes were obtained from Zhang et al. [29]. Each reaction included 12.5 μL of 2× Taq PCR Master Mix (KT201-02; Tiangen, Beijing, China), 8.5 μL of deionized water (Tiangen), 2 μL of DNA, and 1 μL each of upstream and downstream primers, for a total volume of 25 μL. Positive and negative controls were included in each test. All secondary amplifications were visualized under UV light after electrophoresis on a 1% agarose gel mixed with Golden View.

### Nucleotide sequencing and analysis

All positive secondary PCR products were sent to Sangon Biotech Company (Shanghai, China) for sequencing. Genotypes with mutations, including single nucleotide substitutions, deletions, or insertions, were confirmed by DNA sequencing of at least two PCR products. Assemblages and subtypes were identified by the alignment of the nucleotide sequences with known reference sequences for *bg, tpi,* and *gdh* of *G. duodenalis* available in the GenBank database using BLAST and Clustal X.

### Phylogenetic analysis

To assess the genetic relationships among *G. duodenalis* genotypes and previously published reference sequences in GenBank, a phylogenetic analysis was performed. A neighbor-joining tree was constructed using Mega 6 based on evolutionary distances calculated with the Kimura 2-parameter model. The reliability of trees was assessed using a bootstrap analysis with 1,000 replicates.

### Statistical analysis

The χ^2^ test was used to compare the infection rates of *G. duodenalis* at three stray dog shelters in different cities, and differences were considered significant when *p* < 0.05.

## Results

### Occurrence of *G. duodenalis*

In this study, 18 (11.3%) positive specimens were obtained from 159 fecal specimens by nested PCR amplification of the *bg* locus. Among positive specimens, 14 were obtained from dogs with diarrhea and 4 from dogs without diarrhea. The occurrence rates in dogs with and without diarrhea were 35.0% and 3.4%, respectively. The infection rates of dogs with diarrhea were highly significantly different from dogs without diarrhea. (χ^2^ = 29.85, *p* < 0.01). Multilocus sequence typing at *bg*, *gdh*, and *tpi* revealed the presence of *G. duodenalis* assemblages C (13) and D (9). The infection rate in Chengdu was highest (35%; 14/40), followed by Yaan (4.8%; 3/63) and Leshan (1.8%; 1/56) based on amplification of the *bg* locus. The genotypes of all positive specimens are listed in Table 1. Additionally, we found mixed infections in four samples, CD18, CD32, YA16, and YA60.

**Table 1.** Prevalence and distribution of *Giardia duodenalis* by location in Sichuan province, China.

Location (city)	No. samples	No. positive (%)	Genotype (*n*)
Chengdu	40	14 (35.0%)	Assemblage C (8); assemblage D (6)
Yaan	63	3 (4.8%)	Assemblage C (1); assemblage D (2)
Leshan	56	1 (1.8%)	Assemblage D (1)
Total	159	18 (11.3%)	Assemblage C (9); assemblage D (9); assemblage C/D (4)

### Molecular analysis

**Figure 1.**
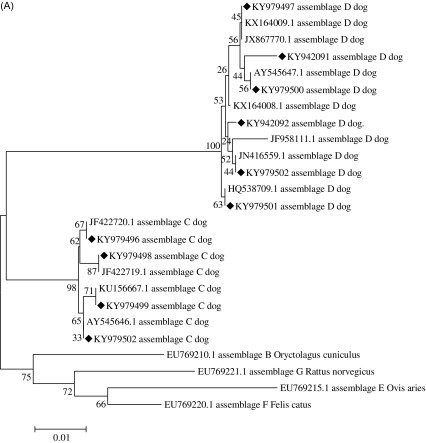
Phylogenetic relationships of *Giardia duodenalis* at the *bg*, *gdh,* and *tpi* loci. The relationships between *G. duodenalis* genotypes identified in this study and other known genotypes deposited in GenBank were inferred by a neighbor-joining analysis of three genetic loci using the Kimura 2-parameter model. Bootstrap values greater than 50% from 1,000 replicates are shown. (A) Tree based on the beta giardin (*bg*) gene. (B) Tree based on the glutamate dehydrogenase (*gdh*) gene. (C) Tree based on the triosephosphate isomerase (*tpi*) gene. Sequences obtained in this study are marked with “◆”.

A total of 18 positive specimens were identified by nested PCR, and a phylogenetic analysis based on *bg*, *gdh*, and *tpi* is summarized in Figure 1. All positive specimens at different loci are listed in Table 2. A multilocus sequencing analysis further identified subtypes of assemblages C and D (see Table 3 for accession numbers).

**Table 2.** Genotypes of *Giardia duodenalis* isolates from dogs in Chengdu, Yaan, and Leshan in Sichuan province, China at the *bg*, *gdh,* and *tpi* loci.

Isolates	*bg*	*gdh*	*tpi*
CD02	C	Neg	Neg
CD17	C	C	C
CD18	D	C	C
CD21	C	C	C
CD23	C	Neg	Neg
CD25	C	C	C
CD27	D	D	Neg
CD28	C	C	C
CD31	C	C	Neg
CD32	D	C	Neg
CD33	D	D	Neg
CD35	D	D	Neg
CD36	D	D	Neg
CD40	C	C	C
YA16	D	C	Neg
YA49	C	C	C
YA60	D	C	C
LS19	D	D	Neg

*Note*: CD represents Chengdu, YA represents Yaan, and LS represents Leshan; Neg represents negative.

**Table 3.** Sequences with SNPs and corresponding accession numbers.

Isolate code	Gene	Genotype	Accession number (new sequence)	Compared accession number
CD23	*bg*	Assemblage D	KY942091	KX164009
YA16	*bg*	Assemblage D	KY942092	KX164008
CD02/17/21	*bg*	Assemblage C	KY979496	JF422720
CD18/27	*bg*	Assemblage D	KY979497	JX867770
CD25/28/36/40	*bg*	Assemblage C	KY979498	JF422719
CD31	*bg*	Assemblage C	KY979499	KU156667
CD23/35	*bg*	Assemblage D	KY979500	KX164009
CD33/YA60	*bg*	Assemblage D	KY979501	HQ538709
YA49/	*bg*	Assemblage C	KY979502	AY545646
LS19	*bg*	Assemblage D	KY979503	JN416559
YA16	*gdh*	Assemblage C	KY942086	EF507635
CD17/Y49	*gdh*	Assemblage C	KY979488	EF507623
CD18/21/25/28/31/32/40/YA60	*gdh*	Assemblage C	KY979489	EF507635
CD27/33	*gdh*	Assemblage D	KY979490	EF507627
CD35	*gdh*	Assemblage D	KY979491	EF507639
LA19	*gdh*	Assemblage D	KY979492	EF507638
CD18	*tpi*	Assemblage C	KY942087	KX014801
CD21/YA49	*tpi*	Assemblage C	KY942088	KX014804
CD28	*tpi*	Assemblage C	KY942089	KF993723
CD40	*tpi*	Assemblage C	KY942090	KP866790
CD17	*tpi*	Assemblage C	KY979493	HG970114
CD25	*tpi*	Assemblage C	KY979494	KX014796
YA60	*tpi*	Assemblage C	KY979495	JN587492

#### Summary of *bg* results

All isolates obtained from dogs were assemblages C and D, including nine that were assemblage C and nine that were assemblage D (Fig. 1A). Among these isolates, two had single nucleotide polymorphisms (SNPs) compared with the reference sequences downloaded from GenBank. For assemblage D, CD23 was similar to KX164009 with two SNPs (substitution: A/C and C/A), and YA16 was similar to KF958111 with one SNP (substitution: C/A).

#### Summary of *gdh* results

Among 16 isolates from dogs, 12 were identified as assemblage C and 4 as assemblage D (Fig. 1B). The YA16 isolate had a SNP compared with the reference sequences downloaded from GenBank. At the *gdh* locus, YA16 was similar to EF507635 with two SNPs (substitution: A/G and T/C).

#### Summary of *tpi* results

Eight isolates were identified as assemblage C at the *tpi* locus (Fig. 1C). Among eight isolates, five had a SNP and two isolates, CD21 and YA49, had the same SNP compared with the reference sequence downloaded from GenBank. For the *tpi* locus, an alignment of all eight assemblage C sequences was generated. CD40 was similar to EU781005 with a SNP (substitution: A/G). CD18 was similar to KX014801 with two SNPs (substitution: G/A and G/A). CD21 and YA49 were similar to KX014804 with a SNP (substitution: A/G). CD28 was similar to KF993723 with a SNP (substitution: A/C).

## Discussion

In China, the prevalence of *G. duodenalis* in dogs has been reported in Heilongjiang [11], Shanghai [27], Guangdong [10], and Henan [19]. Four genotypes, assemblages A, B, C, and D, have been identified in Shanghai in pet dogs [27]. For stray dogs, only assemblages C and D have been identified in Henan [19]. Assemblage E, infecting cattle, has been identified in dogs in Heilongjiang province [11]. In total, five genotypes, i.e., assemblages A, B, C, D, and E, have been found in dogs in China. The prevalent assemblages differ among different areas in China. Five genotypes have been found in Canada. The prevalent genotypes of *G. duodenalis* in dogs in Japan [8], Brazil [4], Poland [17], the Netherlands [14], and England [23] are shown in Table 4.

**Table 4.** Prevalence of *Giardia duodenalis* in different sample sources in China and other countries by amplification of the *bg* locus.

Country/Province	Feces source	Genotypes	Reference
China			
Shanghai	Pet dogs	A, B, C, D	[27]
Guangdong	Pet dogs	A, D	[10]
Heilongjiang	Stray dogs		[11]
	Pet dogs	C, E	[11]
Henan	Stray dogs	C, D	[19]
	Pet dogs	C, D	
Sichuan	Stray dogs	C, D	
Total	Stray dogs	C, D	
	Pet dogs	A, B, C, D	
Other country			
Japan	Pet dogs	C, D	[8]
Brazil	Pet dogs	A, B, C, D	[4]
Poland	Pet dogs	B, C, D	[17]
Spain	Stray dogs	A, B, C, D	[6]
Canada	Stray dogs	C, D, E	[22]
	Pet dogs	A, B, C, D, E	[22]
The Netherlands	Pet dogs	A, C, D	[14]
England	Stray dogs	C, D	[23]
Total	Stray dogs	A, B, C, D, E	
	Pet dogs	A, B, C, D, E	

In most studies of *G. duodenalis* in dogs, assemblages C and D, host-specific genotypes, are considered dominant [10, 19, 27]. Other assemblages, such as assemblage E reported in Heilongjiang [11], have zoonotic potential, to a certain extent. In this study, only the host-specific assemblages C and D were found in stray dogs, similar to previous results in Henan [19]. Moreover, a high frequency of mixed infections of *G. duodenalis* has been reported in previous multilocus analyses [19, 27]. A multilocus genotype method (*tpi*, *gdh,* and *bg* loci) is widely used for the detection of *G. duodenalis* co-infection in humans and animals [3, 19, 26, 27]. In this study, mixed infections of assemblages C and D were also observed, which is consistent with other studies in dogs [19, 27]. Owing to the low levels of contact between people and stray dogs in China, the zoonotic assemblages A and B were not found in this study, which is consistent with another study in Henan [19]. However, the identification of genotype A1 in both a child and his dog in Brazil [24] suggests that the infection in the dog resulted from contact with *G. duodenalis*-infected feces of the owner.

Multilocus sequence typing was used for the genetic characterization of *G. duodenalis* in this study. The *bg*, *gdh*, and *tpi* loci varied with respect to PCR amplification rates, consistent with most previous multilocus typing studies of *G. duodenalis* [2, 19, 25]. The stray dogs in our study did not harbor zoonotic genotypes of *G. duodenalis*, indicating a minimal role in zoonotic transmission in Sichuan province, China.

The results obtained in this study demonstrate that genetic assemblages C and D of *G. duodenalis* are present in stray dogs in Sichuan province, China. Zoonotic genotypes (assemblages A and B) were not found, suggesting that these genotypes are not prevalent in stray dogs in Sichuan province, China. Moreover, new subtypes were identified. Nevertheless, *G. duodenalis* is a prevalent protozoan parasite, and although zoonotic assemblages were not found in stray dogs in this study, potential transmission should not be overlooked. Certain measures should be taken to reduce the possibility of intraspecific transmission.

## Conflict of interest

The authors declare that they have no conflict of interest.
